# Aerobic exercise modulates intracortical inhibition and facilitation in a nonexercised upper limb muscle

**DOI:** 10.1186/2052-1847-6-23

**Published:** 2014-06-21

**Authors:** Amaya M Singh, Robin E Duncan, Jason L Neva, W Richard Staines

**Affiliations:** 1 Department of Kinesiology, University of Waterloo, 200 University Avenue West, Waterloo, ON N2L 3G1, Canada

**Keywords:** Aerobic exercise, Primary motor cortex, Transcranial magnetic stimulation, Intracortical inhibition, Intracortical facilitation

## Abstract

**Background:**

Despite growing interest in the relationship between exercise and short-term neural plasticity, the effects of exercise on motor cortical (M1) excitability are not well studied. Acute, lower-limb aerobic exercise may potentially modulate M1 excitability in working muscles, but the effects on muscles not involved in the exercise are unknown. Here we examined the excitability changes in an upper limb muscle representation following a single session of lower body aerobic exercise. Investigating the response to exercise in a non-exercised muscle may help to determine the clinical usefulness of lower-body exercise interventions for upper limb neurorehabilitation.

**Methods:**

In this study, transcranial magnetic stimulation was used to assess input–output curves, short-interval intracortical inhibition (SICI), long-interval intracortical inhibition (LICI) and intracortical facilitation (ICF) in the extensor carpi radialis muscle in twelve healthy individuals following a single session of moderate stationary biking. Additionally, we examined whether the presence of a common polymorphism of the brain-derived neurotrophic factor (BDNF) gene would affect the response of these measures to exercise.

**Results:**

We observed significant increases in ICF and decreases in SICI following exercise. No changes in LICI were detected, and no differences were observed in input–output curves following exercise, or between BDNF groups.

**Conclusions:**

The current results demonstrate that the modulation of intracortical excitability following aerobic exercise is not limited to those muscles involved in the exercise, and that while exercise does not directly modulate the excitability of motor neurons, it may facilitate the induction of experience-dependent plasticity via a decrease in intracortical inhibition and increase in intracortical facilitation. These findings indicate that exercise may create favourable conditions for adaptive plasticity in M1 and may be an effective adjunct to traditional training or rehabilitation methods.

## Background

The benefits of exercise on brain function have been widely documented. However, little is known about the direct effects of exercise on motor cortical excitability. In clinical settings, aerobic exercise is commonly prescribed to improve cardiovascular function following brain injury and can successfully improve aerobic capacity in neurological patient populations [1-4]. In addition to secondary cardiovascular disease prevention and improved quality of life, emerging evidence suggests exercise may also promote beneficial cortical adaptations. Plasticity in the motor cortex is among the primary goals of rehabilitation programs following brain injury, and much attention has been focused on the ability of exercise to act as a potential primer for subsequent task-specific changes in cortical excitability associated with learning-based rehabilitation. However, the mechanisms underlying such modulation are not yet known. While chronic physical activity is associated with increased metabolic capacity and increased angiogenesis in the primary motor cortex (M1) [5,6], little is known about how aerobic exercise modulates cortical excitability, and the effects of an acute bout of aerobic exercise on the motor cortex are unclear.

Recently, pedaling exercise has been shown to decrease intracortical inhibition in the leg region of M1 [7], which suggest that such an intervention may be effective in increasing excitability. However, in clinical settings, spasticity and muscle weakness are seen frequently in the upper limbs, particularly following a stroke. Up to 85% of stroke patients present with hemiparesis of the upper limbs [8,9], and thus the upper limb musculature is often the focus of rehabilitation. Yet, the majority of clinical aerobic exercise interventions, such as walking, running and cycling, predominantly involve the lower limbs. Pedaling exercise is frequently used in rehabilitation settings for patients who exhibit gait disturbances, or who present with balance or stability issues. Recent evidence indicates that acute cycling modulates intracortical inhibition in the cortical representations of active muscles; however, it is not known if this response is limited to muscles involved in the exercise or if such responses can be observed in nonexercised limbs. Here, we use transcranial magnetic stimulation (TMS) to probe both the excitability of descending tracts of nonexercised muscles following exercise and the intracortical inhibitory and facilitatory networks within M1. We assessed the effect of aerobic exercise on corticospinal excitability by using single pulses to generate a stimulus–response (S-R) curve at varying intensities. Three paired-pulse paradigms were used to probe the effect of exercise on the intracortical networks within M1: short-interval intracortical inhibition (SICI), long-interval intracortical inhibition (LICI) and intracortical facilitation (ICF).

The primary aim of this study was to investigate the effects of a brief session of lower-limb aerobic exercise on the cortical excitability of an upper-limb muscle representation. In addition, we investigated whether the presence of a common single nucleotide polymorphism (a valine-to-methionine substitution at codon 66, or Val66Met) of the brain-derived neurotrophic factor (BDNF) gene would impact the cortical response to exercise. BDNF is a growth factor secreted by the brain that is critical for the growth and survival of neurons and plays a key role in the development of long-term potentiation (LTP). The Val66Met polymorphism is associated with decreased activity-dependent BDNF release and has been linked to diminished motor cortical plasticity, with Met carriers displaying decreased task-related M1 activation [10], reduced responses to the induction of experience-dependent plasticity [11], and impaired synaptic transmission [12]. Thus, we examined whether genetic variability in the BDNF gene would affect the response of M1 to aerobic exercise. We found that while the input–output curve and LICI were not significantly affected by exercise, lower-limb exercise induced a significant decrease in SICI and increase in ICF in a non-exercised muscle. None of the above measures were significantly affected by the presence of the BDNF polymorphism. These findings may have important implications for the use of aerobic exercise in treating upper limb motor deficits.

## Methods

### Participants

Twelve young, healthy, moderately active individuals were recruited (7 males; 1 left-handed, 11 right-handed; average age = 28 years). All participants had prior experience with TMS. Informed consent was obtained from all participants prior to undergoing the experimental protocol and they were screened for any contraindications to TMS using a standard screening form. All experimental procedures received clearance from the University Of Waterloo Office Of Research Ethics.

### Exercise protocol

Heart rate (HR) and rate of perceived exertion (RPE) were collected at rest prior to exercise. During exercise, heart rate was monitored using a wrist-mounted heart rate monitor. Participants were instructed to work at 65-70% of their age-predicted maximal heart rate [average = 125-135 beats per minute (bpm)]. After a brief warm-up to elevate HR into the target zone, participants performed 20 minutes of continuous stationary biking on a recumbent bicycle in an isolated room. The duration and intensity were intended to mimic a standard aerobic workout. HR was carefully monitored and maintained throughout the session. Participants were seated comfortably with their feet strapped to the pedals and their backs against the backrest. RPE was verbally reported using the modified Borg scale every five minutes, and HR was continuously monitored throughout the exercise period. Instructions were given to work at a moderate intensity (RPE of 3–4), and participants could adjust either the pedaling resistance or the rate of pedaling to maintain the target heart rate. All participants reported intensity rates in the moderate range, with no individual exceeding an RPE of 4. The experimenters remained with the participant throughout the exercise and ensured that arms were resting comfortably by their sides and not gripping the handlebars during the session. The arms, and particularly the forearms, remained stationary during pedaling exercise. Participants were given free access to water. Immediately following exercise completion, participants returned to the TMS testing room for the collection of post-exercise measures. Post 1 occurred immediately following exercise and lasted approximately 10 minutes. Participants then rested in an upright chair in a quiet room for the remainder of the rest period. Post 2 was collected 30 minutes following exercise completion. In all cases, heart rate had returned to resting or near-resting levels (within 5 bpm) by the 30 minute mark post-exercise.

### BDNF genotyping

The brain-derived neurotrophic factor (BDNF) Val66Met polymorphism (rs6265) was genotyped from saliva samples by qPCR on an ABI7500 using a TaqMan SNP genotyping assay (Applied Biosystems) with 10 ng of saliva genomic DNA isolated by standard procedures from anonymized samples. Random duplicate analyses showed 100% concordance with runs.

### TMS protocol

Focal TMS was performed using a MagPro × 100 stimulator (Medtronic, Minneapolis, MN, USA) and a ‘figure of eight’ coil (MCF-B65; 70 mm). BrainSight Neuronavigation (Rogue Research, Canada) was used to guide the placement of the coil to the target motor region using a template MRI for all participants. Anatomical co-registration was performed prior to baseline collection and subsequent coil positioning was tracked using reflective markers affixed to custom-fitted glasses. The coil was placed at a 45° angle to the mid-sagittal line to induce a posterior to anterior current in the underlying neural tissue. EMG recordings of motor-evoked potentials (MEPs) were obtained using surface electrodes placed over the right extensor carpi radialis muscle (ECR). Raw EMG signals were recorded and stored in a customized Labview (National Instruments, Austin, TX, USA) program for offline analysis. The motor hotspot of the right ECR muscle was identified as the left M1 location that consistently elicited a maximal MEP in the resting muscle, as assessed by EMG amplitude, while producing a visible muscle twitch. The resting motor threshold (RMT) was determined by the stimulation intensity required to elicit a peak-to-peak MEP amplitude of >50 μV on 5 out of 10 trials. After localization of the hotspot, a stimulus–response curve was generated by assessing the cortical response to single-pulse TMS at a range of intensities. Ten single pulses were delivered with a minimum of 2-second intervals at stimulus intensities of 100%, 110%, 120%, 130%, and 140% of RMT. Three paired-pulse measures were also assessed using the following parameters for the conditioning stimulus (CS), test stimulus (TS) and inter-stimulus interval (ISI): a) SICI (CS = 80% and TS = 120% of RMT, 2.5 ms ISI); b) LICI (CS = 120% and TS = 120% of RMT, 100 ms ISI); and c) ICF (CS = 80% and TS = 120% of RMT, 12 ms ISI). Ten pairs of stimuli were delivered in each paired-pulse protocol with an ISI of 2 seconds between stimulus pairs. Thus, the following four measures were randomized across participants but the order remained consistent throughout each individual experiment: i) S-R curve, ii) SICI, iii) LICI, and iv) ICF. Measures were collected just prior to exercise, immediately following exercise, and again 30 minutes following exercise completion.

### Statistical analysis

In all paired-pulse measures, the degree of inhibition or excitation was normalized to the single pulse amplitude at 120% RMT for each timepoint. Participants in whom intracortical inhibition could not be induced pre-exercise using standard protocols were excluded from the corresponding analysis. For SICI and ICF, the average amplitude elicited by the conditioned stimulus was expressed as a percentage of the average unconditioned MEP amplitude at 120%. For LICI, the amplitude of the MEP evoked by the test pulse was expressed as a percentage of the conditioning stimulus amplitude, and the average of 20 trials was taken. For the S-R curves, 10 MEPs were averaged at each intensity and the average values were compared. To assess changes in resting single-pulse excitability within the S-R curves, a two-way repeated measures ANOVA was conducted with time (pre, post 1 and post 2) and stimulus intensity (100%, 110%, 120%, 130% and 140% RMT) as factors. Paired-pulse measures were analyzed using three separate one-way repeated measures ANOVAs for SICI, LICI and ICF data using time as the main factor. Post hoc testing was performed using Tukey’s HSD. To test the effect of the BDNF polymorphism, subjects were divided into Met carriers (n = 6) or non-Met carriers (n = 6). A mixed 2 × 3 × 5 ANOVA was conducted to assess differences in S-R curves between Met carriers and non-Met carriers using stimulus intensity and time as the within-subjects factors and genotype as the between-subjects factor. The response to paired-pulse measures within each group was assessed using separate two-way mixed ANOVAs for SICI, LICI, and ICF, with time as the within-subjects factor and genotype as the between-subjects factor. Significant main effects in the ANOVA were followed with post hoc tests using Tukey’s HSD. The significance level for all tests was set at p < 0.05.

## Results

**Figure 1:**
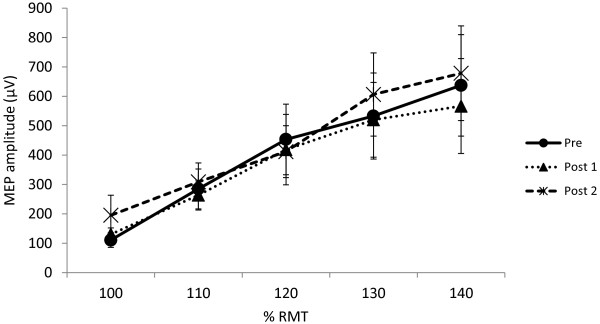
**Recruitment curves before and after exercise.** Stimulus–response curves pre- and post-exercise in response to stimulation at increasing percentages of RMT (n = 12). Bars represent SEM. (* = p < 0.05).

Although EMG was not collected continuously during the exercise session, offline testing was conducted to monitor upper limb muscle activity during an identical biking task. There was no detectable muscle activity in right ECR, flexor carpi radialis (FCR), or first dorsal interosseous (FDI) during biking. For all measures, pre-exercise responses were taken as baseline values. Figure 1 displays the S-R curves, with the average MEP amplitude evoked in response to varying stimulus intensities at each timepoint. Not surprisingly, a two-way repeated measures ANOVA showed a significant main effect of intensity (F_4, 44_ = 9.70, p < 0.001, Figure 1). Post-hoc testing using Tukey’s HSD revealed that MEP amplitude differed significantly between 100% RMT and 120%, 130% and 140% RMT. In addition, evoked responses at 110% differed significantly from those at 130% and 140%. There was no main effect of timepoint relative to exercise (F_2, 22_ = 1.59, p = 0.23) and no interaction between intensity and timepoint (F_8, 88_ = 1.11, p = 0.36).

**Figure 2:**
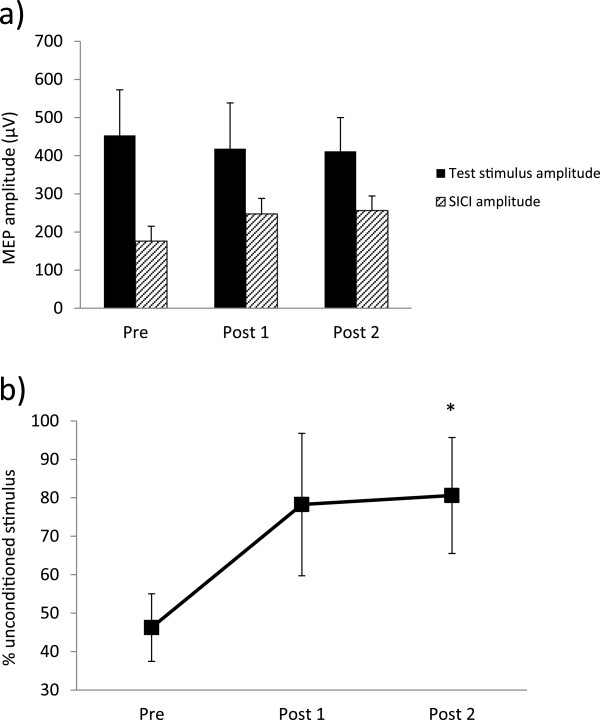
**Modulation of SICI following exercise. (a)** Induction of SICI across all participants (n = 11) at each timepoint and **(b)** % of test stimulus amplitude. Unconditioned single pulse amplitudes at 120% RMT are compared to conditioned stimulus amplitudes. Bars represent SEM. Asterisks indicate values significantly different from pre-exercise (p < 0.05).

**Figure 3:**
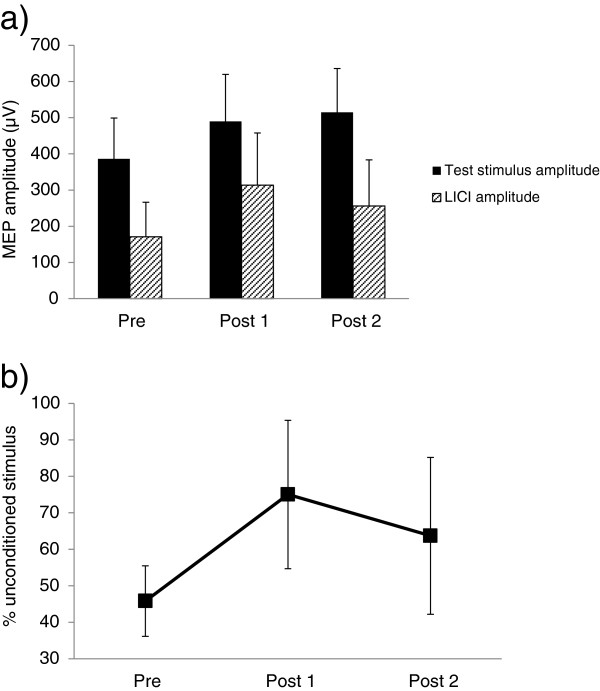
**Modulation of LICI following exercise.** Induction of LICI across all participants (n = 11) at each timepoint **(a)** and % of test stimulus amplitude **(b)**. Unconditioned single pulse amplitudes at 120% RMT are compared to conditioned stimulus amplitudes. Bars represent SEM.

**Figure 4:**
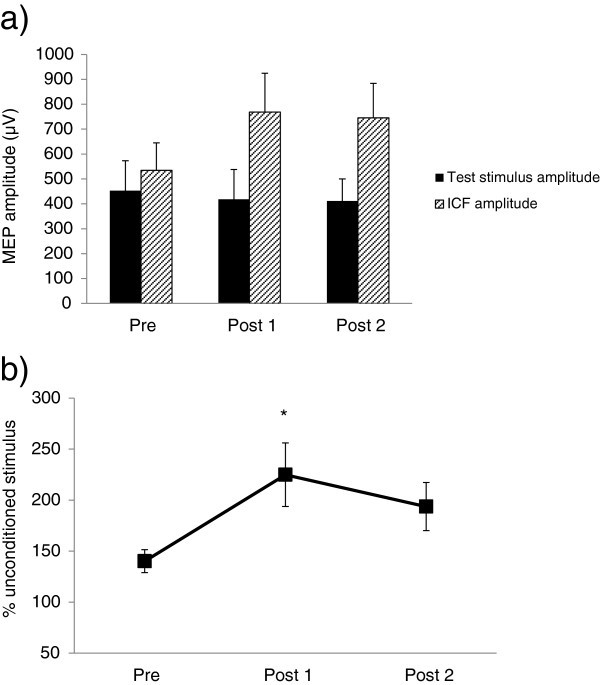
**Modulation of ICF following exercise.** Induction of ICF across all participants (n = 12) at each timepoint **(a)** and % facilitation of test stimulus **(b)**. Unconditioned single pulse amplitudes at 120% RMT are compared to conditioned stimulus amplitudes. Bars represent SEM. Asterisks indicate values significantly different from pre-exercise (p < 0.05).

Average paired-pulse responses across all subjects are shown in Figures 2, 3 and 4. The above-noted exclusion criteria resulted in one participant being removed from the SICI analysis and one participant from the LICI analysis. Figure 2a displays the consistency of SICI induction across each timepoint. Results of a one-way repeated measures ANOVA showed that following exercise, SICI was significantly decreased (F_2, 20_ = 4.30, p = 0.028, Figure 2b). Prior to exercise, SICI induced an average (±standard error) of 53.8 ± 8.8% inhibition of the unconditioned stimulus. Immediately after exercise (post 1), SICI levels decreased to 21.8 ± 18.5% and remained at 19.4 ± 15.1% 30 minutes following exercise (post 2). Post-hoc testing using Tukey’s HSD revealed a significant decrease in SICI from pre to post 2. Results from the LICI analysis demonstrate a similar trend (Figure 3): pre-exercise levels of LICI showed 54.2 ± 9.6% inhibition of test stimulus amplitude. Following exercise, this decreased to 25.0 ± 20.3% and increased slightly to 36.3 ± 21.5% at post 2; however, these differences were not statistically significant (F_2, 20_ = 1.36, p = 0.28, Figure 3b). Correspondingly, a one-way repeated measures ANOVA of ICF revealed that following exercise, ICF was significantly elevated (F_2, 22_ = 5.29, p = 0.013, Figure 4b). Baseline values showed a 140.1 ± 11.2% increase relative to unconditioned stimulus amplitudes. At post 1, ICF values increased to 224.8 ± 31.1% of unconditioned test pulses. ICF levels remained elevated at post 2, with an average of 193.7 ± 23.6% facilitation. Post-hoc testing revealed significant differences between pre and post 1, and while ICF levels remained elevated at post 2 relative to pre, this difference was not statistically significant.

**Figure 5:**
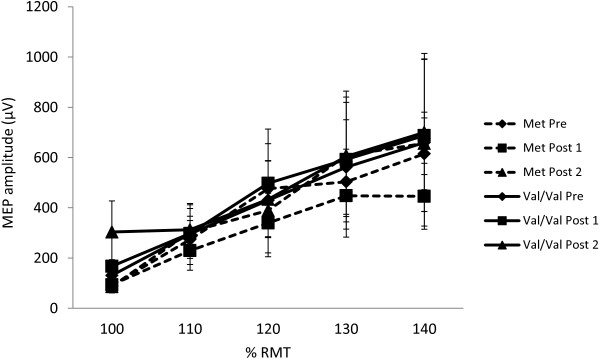
**Effect of BDNF genotype on recruitment curves.** Group differences between Met carriers (n = 6) and non-Met carriers (n = 6) in S-R curve outputs at each timepoint. Bars represent SEM.

Results from BDNF genotyping indicated that six of twelve subjects were Met carriers (two homozygous and four heterozygous). Results from a 2 × 3 × 5 mixed ANOVA performed on S-R curves indicated no significant differences between Met carriers and Val/Val subjects in single-pulse excitability at any timepoint or any stimulus intensity (F_1, 7_ = 0.14, p = 0.71, Figure 5).

**Figure 6:**
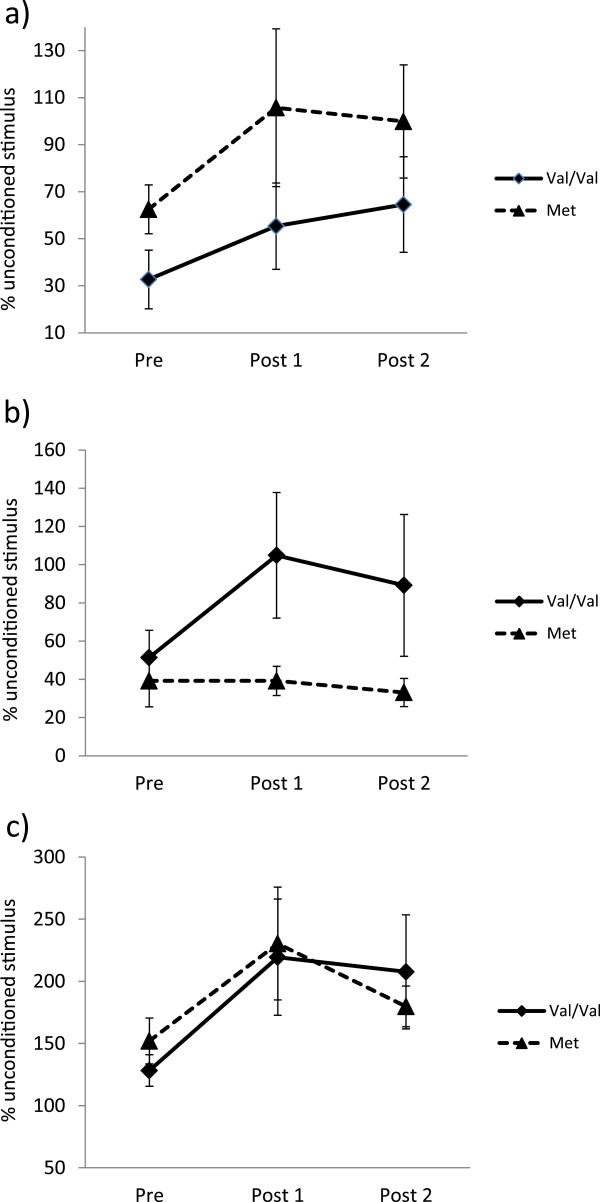
**Effect of BDNF genotype on intracortical inhibition and facilitation.** Group differences between **a)** Met carriers (n = 6) and non-Met carriers (n = 5) for SICI; **b)** Met carriers (n = 5) and non-Met carriers (n = 6) for LICI, and **c)** Met carriers (n = 6) and non-Met carriers (n = 6) for ICF. Bars represent SEM.

Results from separate two-way mixed ANOVAs revealed no main effect of BDNF for SICI (F_1, 9_ = 2.71, p = 0.13), LICI (F_1, 9_ = 2.66, p = 0.14), or ICF (F_1, 10_ = 0.00035, p = 0.95), and no BDNF × time interaction for SICI (F_2, 18_ = 0.3, p = 0.74), LICI (F_2, 18_ = 1.3, p = 0.30), or ICF (F_2, 20_ = 0.5, p = 0.62) (Figure 6).

## Discussion

The aim of this study was to test whether the modulation of the cortical excitability of a specific muscle representation in M1 following aerobic exercise is dependent on the involvement of that muscle in the exercise itself. Specifically, we sought to investigate whether aerobic exercise involving the lower limbs could modulate upper limb motor cortical excitability and also to determine the time course of this modulation and potential mechanisms that contribute to it. Thus, both S-R curves and paired-pulse measures of SICI, LICI and ICF were used to probe the excitability changes in a wrist extensor muscle following a single session of stationary biking. Immediately after exercise completion, there was a significant decrease in short-interval intracortical inhibition and a significant increase in intracortical facilitation. Immediately after exercise completion, there was a significant increase in intracortical facilitation, and a significant suppression of SICI was evident at 30 minutes post-exercise. While LICI displayed a similar trend to SICI, in this case the decrease in inhibition was not statistically significant. In contrast, the S-R curves indicate that the resting motor threshold was not modulated by exercise. There were no significant differences observed in MEP amplitudes pre- and post-exercise at any intensity. Thus, resting motor thresholds of inactive muscles appear unchanged by exercise. However, the current results indicate that aerobic activity using the lower limbs causes an immediate and sustained modulation of intracortical facilitation and inhibition of an upper limb muscle. Such excitability changes are a necessary precursor to the relatively more permanent changes in synaptic strength seen in the processes of long-term potentiation (LTP) and long-term depression (LTD). It is likely that the altered excitability state of these interneuronal pools will render them more receptive to strategies aimed at inducing plasticity, such as skilled motor training or targeted rehabilitation, when they are preceded by an exercise session. Furthermore, interventions that directly target the mechanisms of LTP/LTD, such as repetitive theta-burst stimulation (TBS), may benefit from the addition of exercise. It should be noted, however, that the benefits of such interventions will not necessarily be additive. The emerging principles of homeostatic metaplasticity suggest that the probability of LTP induction depends on prior synaptic activity and that when LTP has been recently induced, subsequent facilitatory interventions will be suppressed or even reversed in order to maintain a balance between LTP and LTD [13-15].

As indicated, previous research has demonstrated a decrease in SICI in exercising muscles [7]. The current results extend this finding to non-exercised muscles and indicate that such changes are not a direct consequence of preceding muscle activity. These results are in line with the findings of Takahashi et al. [16], who report that lower limb resistance exercise influences cortical excitability in nonexercised hand muscles. Takahashi and colleagues [16] propose several potential mechanisms for their findings, including facilitatory cortical pathways between synergistic arm and leg representations, and a spread of cortical excitability from active muscles to non-active muscles in proximal M1 areas. Neither of these possibilities can be ruled out here. However, the observed changes were seen at the motor hotspot of the ECR and not on the periphery of the representation, which would indicate a modulation of the ECR representation itself. Furthermore, the lack of an effect on single-pulse amplitude after exercise argues against a spread of excitability from active muscle representations. Nor do the current results address the contribution of spinal circuits. Although decreases in H-reflex amplitude following prolonged aerobic exercise have been reported in lower limb muscles [17], upper limb muscles are unaffected, indicating that such changes do not represent a generalized decrease in spinal excitability but rather are specific to those muscles involved in locomotion [18]. Additionally, one would expect a change in spinal networks to be reflected in the single pulse excitability. In contrast, emerging evidence suggests that aerobic exercise is uniquely suited to cause a more generalized increase in intracortical excitability following exercise [19-23]. Indeed, a model of a more widespread neural effect of exercise is well-supported. Chronic physical activity is associated with increased activation of regions as diverse as the superior parietal cortex and the dentate gyrus [19], and can modulate everything from pain perception [20] to mood [21]. Further, it is clear that lower limb aerobic exercise can affect vascular functioning in upper limb muscles [22]. Indeed, a single bout of moderate intensity stationary biking can induce a 20% increase in global cerebral blood flow (CBF) [23]. Yet, it has been hypothesized that with limited metabolic resources, exercise may upregulate those regions involved with maintaining exercise [24] which, it is assumed, includes movement-related cortical regions such as M1. Such a global response could be mediated by the supplementary motor area or the prefrontal cortex, both of which have shown increased activity with exercise [25-27].

### Role of GABA and clinical significance

The mechanisms that may underlie a more widespread response to exercise are not entirely clear; however, there is strong evidence that exercise can modulate neurotransmission. Acute aerobic exercise has been shown to upregulate the activity and/or release of serotonin (5HT) [28,29], dopamine (DA) [28,30,31], and norepinephrine (NE) [31,32], all of which can modulate the excitability of M1 neurons [33-36]. Exercise-induced increases in blood lactate have shown corresponding increases in M1 excitability [37], while increased uptake of the trophic factor insulin-like growth factor 1 (IGF-1) appears to mediate an increase in neuronal sensitivity and firing rates post-exercise [38]. Both the time course of the exercise-induced changes in excitability and the optimal exercise parameters for stimulating the release of neurotrophic factors remain under investigation. While the potential contribution of such excitatory neurotransmitters cannot be discounted here, the current results point to modulations in GABA (γ-aminobutyric acid) as a primary outcome of exercise. GABA is the principal inhibitory neurotransmitter in the CNS and exerts its effects via multiple receptors, particularly in cortical inhibitory networks. SICI is thought to be mediated by GABA_A_ receptors [39], which are ligand-gated chloride channels, while LICI is believed to activate GABA_B_ receptors [40], which are coupled to G-protein complexes that activate downstream K^+^ ion channels. Although the cortical mechanisms of ICF are not fully understood, it appears to be mediated by glutamatergic interneurons, and possibly NMDA receptors [41,42]. While both LICI and SICI directly affect the excitability of corticospinal neurons, there are also interactions between them, as LICI appears to reduce SICI, likely via GABA-mediated inhibition of GABA-release [39,43]. The current results indicate that SICI is more sensitive to the effects of aerobic exercise than LICI. This is perhaps not surprising given that there appears to be little correlation between SICI and LICI measures [40,43]. Indeed, it has been suggested that GABA_A_ and GABA_B_ receptors may differ in their activation thresholds, with GABA_A_ receptors requiring greater levels of exposure to the neurotransmitter [44]. Another potential reason for this disconnect is the variation in test stimulus intensities, in that SICI and ICF both employ a subthreshold conditioning pulse that is assumed to activate intracortical connections, while LICI requires two suprathreshold pulses, and may therefore be activating a different pool of neurons.

Such intracortical networks are critical to the modulation of cortical output and are implicated in cortical plasticity and reorganization [45]. The release of GABA at inhibitory synapses directly modulates the excitability of pyramidal cells and the current results suggest this process may be sensitive to exercise. There is limited information available on GABA levels immediately following exercise; however, a downregulation of GABA signalling on baroreceptor neurons is thought to contribute to post-exercise hypotension [46]. Further, mRNA levels of a key GABA_A_ receptor subunit are reduced after only 3 days of exercise training [47]. Meeusen et al. [31] report up to a 76% increase in striatal GABA levels following 60 minutes of treadmill running, although their data did not reach statistical significance. There are considerable clinical implications of an exercise-induced modulation of GABA activity. Decreases in GABA are critical for motor learning and M1 plasticity [48,49]. Indeed, excessive inhibition is a key cause of post-stroke motor impairment [50-52]. GABA blockade removes tonic inhibition and promotes plasticity [53], and indeed, a decrease in GABA levels is key to functional recovery after stroke [53-55]. It is clear that motor reorganization following a brain injury is dependent on functional plasticity. As GABA levels were not directly measured in this study, we cannot determine whether exercise results in changes in GABA release, uptake, or activity, or alters the sensitivity of GABA_A_ receptors. However, these results indicate that there is a reduction in short-interval intracortical inhibition following aerobic exercise, which is likely mediated by exercise-induced changes in GABA_A_ activity.

Our results, taken together with previously observed increases in excitatory neurotransmission, indicate that the net effect of exercise appears to be a decrease in M1 inhibition that may facilitate the induction of plasticity. In the current study, these effects are seen immediately after exercise and persist at 30 minutes after exercise completion. Thus, it is possible that the intracortical network changes seen here are a necessary precursor for cortical plasticity, and that exercise creates the conditions under which more permanent plastic changes may occur. The current results indicate that in non-active muscles, exercise alone does not directly affect the resting motor threshold of pyramidal cells, but instead modulates the balance of inhibitory and excitatory inputs to these cells. This is supported by the findings of Smith et al. [23], who despite observing a global increase in CBF following exercise, did not see an observable modulation in M1 until a subsequent motor task was performed. In addition, McDonnell et al. report no changes in MEP amplitude in the FDI muscle following cycling exercise, but instead demonstrate that the effects of theta-burst stimulation (TBS) are potentiated when preceded by exercise [56]. Thus, while exercise may not modulate CST excitability in and of itself, it can potentially create favourable conditions for the induction of cortical plasticity with subsequent motor training. Indeed, aerobic exercise training has been shown to improve motor arm function after stroke [57,58], and the combination of exercise and skilled motor training improves motor recovery to a greater extent than training alone [59].

Thus, in this context, it is perhaps not surprising that the paired-pulse measures here do not correlate with the single-pulse data, in which we observed a decrease in SICI and an increase in ICF, but no concomitant increases in single-pulse MEP amplitude. This would seem to indicate that there is not a direct correlational relationship between these two measures. Previous studies have reported a similar disconnect between single and paired-pulse measures of CST excitability [33,43,60]. Indeed, Ilic et al. [33] propose that single and paired-pulse measures may reflect substantially different mechanisms. The final corticospinal output reflects the summation of all inhibitory and excitatory inputs to the descending neuron, and can be influenced by many factors, both cortical and subcortical. The paired-pulse measures taken here reflect the activity of particular cortical interneuron pools whose activity may be modulated by exercise, but which are only one of a multitude of inputs on the descending motor neuron.

### BDNF

As a neurotrophic factor, the relationship between acute exercise and BDNF is not clear. Although increases in levels of serum BDNF have been reported following acute aerobic exercise [61-65], BDNF is known to exert its effects primarily over longer time frames and is correlated with the induction of LTP and postsynaptic modification [66]. Thus, it is unlikely that BDNF levels significantly influenced the response to exercise seen here.

Although not the principal aim of this study, we were interested in exploring the relationship between a relatively common single nucleotide polymorphism of the BDNF gene and exercise-related changes in cortical excitability. The valine-to-methionine substitution at codon 66 of the BDNF gene occurs in approximately 30% of the population [67] and is associated with decreased activity-dependent BDNF release and impaired synaptic and cortical plasticity [10,11,68]. Here, as in the majority of the literature, Val/Met and Met/Met individuals were grouped together and compared to Val/Val subjects. There was no difference between the groups in the S-R curves before or at either time point following exercise. Nor was there any interaction between BDNF and time, indicating that genotype did not influence the response to exercise. Previous studies investigating the response to facilitatory intermittent TBS have reported impairments [68,69] or no difference [70,71] in Met carriers, but methodological differences prevent direct comparisons of these studies. While the current sample size is smaller than in the above studies, a key difference is their use of a technique known to induce LTP-like plasticity. The neurological response to exercise is not well-understood, and as such it is not clear how such changes relate to the mechanisms underlying LTP. In the current study, two interesting trends are evident, in that Met carriers, on average, display a complete abolition of SICI following exercise (Figure 6a). Secondly, Met carriers appear to be more resistant to the modulation of LICI following exercise (Figure 6b). Indeed, the lack of response in this group is likely the reason the overall group effect for LICI failed to reach significance. While preliminary, these trends suggest modulations in GABA_B_ receptor activity or sensitivity may contribute to the impaired short-term plasticity frequently observed in Met carriers, and warrants further investigation with a larger subject pool. Our aim was to investigate whether Met carriers would still display these exercise-induced effects, and these results suggest that Met carriers display no impairment in the response to exercise-induced modulations in SICI and ICF.

## Conclusions

The present results suggest that lower-body focused aerobic activity can modulate cortical excitability in an upper limb muscle and that at the cortical level, exercise may prime the motor cortex for the induction of plasticity. While these findings have potential clinical utility, further research will be required to determine how the relationship between exercise and cortical excitability may be altered by disruptions to the balance of cortical inhibition and facilitation following a brain injury, and how the response to exercise is affected by characteristics such as the location, severity and type of brain injury. However, the current findings support the use of aerobic training as an adjunct to traditional rehabilitation methods. A potential limitation of this study is that EMG data was not collected during the exercise session. Although a lack of upper limb activity was confirmed with offline EMG, there nevertheless remains the slight possibility of upper limb muscle activation during the biking session. A second limitation of this study is the investigation of only one upper limb muscle. However, given that changes in upper limb excitability following lower body aerobic exercise are not well-studied, our goal was to create a comprehensive profile of excitability changes that would be sensitive to modulations in both motor neurons and interneurons. This, combined with the time-sensitive nature of the post-exercise measures, made it difficult to test additional muscles. The generalizability of our findings across the upper limb is an interesting direction for future studies.

## Competing interests

The authors declare they have no competing interests.

## Authors’ contributions

AMS carried out the study and data analysis and drafted the manuscript. RD carried out the genetic analysis and assisted with manuscript preparation. JLN assisted with the study design, data collection, and data analysis. WRS helped to conceive the study, wrote the collection and analysis programs, and critically revised the manuscript. All authors read and approved the final manuscript.

## Pre-publication history

The pre-publication history for this paper can be accessed here:

http://www.biomedcentral.com/2052-1847/6/23/prepub
